# Green transformational leadership and its prediction of employees’ green behavior: A dataset from the petroleum retail sector in Vietnam

**DOI:** 10.1016/j.dib.2026.112850

**Published:** 2026-05-16

**Authors:** Thuy Phuong Thi Do, Ha Thu Nguyen, Tuan Dinh Tran

**Affiliations:** a Faculty of Accounting, Thai Nguyen University of Economics and Business Administration, Thai Nguyen, Vietnam; b University of Transport Technology, 54 Trieu Khuc, Thanh Liet, Ha Noi, Vietnam

**Keywords:** Green transformational leadership, Employees’ green behavior, Green work engagement, Meaningful work, Vietnam

## Abstract

This dataset investigates the complex interplay between Green Transformational Leadership, Green Work Engagement, Meaningful Work, and Employees’ Green Behavior in Vietnam’s petroleum retail sector. Data were collected through a structured questionnaire distributed to employees of petroleum retail companies across Northern Vietnam, yielding 347 valid responses using a combination of convenience and snowball sampling techniques. This dataset enables researchers to explore relationships among Green Transformational Leadership, Green Work Engagement, Meaningful Work, and Employees’ Green Behavior, and supports replication and comparative studies in similar contexts.

Specifications Table

**Table:** 

Subject	Social Sciences
Specific subject area	Leadership, Organizational Behaviour, Petroleum Retail Sector, Human Resource Management.*.*
Type of data	Table, Figure Raw.
Data collection	The data for this study was gathered directly from October 2024 and February 2025 through a combination of convenience sampling and snowball sampling techniques. Respondents were asked to complete a structured questionnaire comprising five demographic items and 24 items measuring key constructs: Green Transformational Leadership, Employees’ Green Behavior, Green Work Engagement, and Meaningful Work. All items were rated on a five-point Likert scale. Prior to participation, respondents were informed about the study’s objectives and its significance. Only individuals currently working in the petroleum retail sector and aged 18 or above were considered eligible for this study*.*
Data source location	Northern of Vietnam*.*
Data accessibility	Repository name: Mendeley Data Data identification number: 10.17632/vx8wbjd828.2 Direct URL to data: https://data.mendeley.com/datasets/vx8wbjd828
Related research article	*None*

## 1 Value of the Data

• This dataset provides essential insights into Employees’ Green Behavior within Vietnam’s petroleum retail sector, a critical yet understudied sector in the context of environmental sustainability.
• The survey explores the influence of Green Transformational Leadership, Green Work Engagement, and Meaningful Work on Employees’ Green Behavior, offering a multidimensional understanding of the drivers of pro-environmental behavior in the workplace.
• The dataset enables the examination of relationships between Green Transformational Leadership and Employees’ Green Behavior, as well as the potential roles of Green Work Engagement and Meaningful Work in these relationships.
• The findings can inform policymakers and organizational leaders seeking to promote green practices among employees, especially within energy-related sectors in emerging economies.
• The study is situated in an emerging market context and a strategically vital industry, and it presents opportunities for cross-country and cross-industry comparative research. It can serve as a reference point for researchers from developed economies or other sectors aiming to replicate or extend the study’s findings.
• Previous research has mainly examined the direct link between Green Transformational Leadership and Employees’ Green Behavior or explored this relationship through alternative mediators [[1], [2], [3]]. Furthermore, empirical evidence on how Green Transformational Leadership fosters Employees’ Green Behavior within Vietnam’s petroleum retail sector remains scarce, and this dataset addresses this gap with original, high-quality data from an emerging country.

## 2 Background

This dataset provides empirical insights into Green Transformational Leadership, Green Work Engagement, Meaningful Work, and Employees’ Green Behavior relationships within Vietnam’s petroleum retail sector. In Vietnam, petroleum is a commodity with a vital role and position of strategic significance in ensuring stability and maintaining major balances of the economy, especially ensuring national energy security, serving socio-economic development, and serving people and businesses [4]. As a critical component of the national energy supply chain, the petroleum retail sector enables the smooth functioning of logistics and trade across both urban and rural regions.

In recent years, Vietnam's rapid economic development has significantly increased energy consumption and CO₂ emissions. According to the Vietnam Energy Outlook - Roadmap to Net Zero (2024), the country must peak its carbon emissions by 2030 to meet its 2050 net-zero target, highlighting the urgent need for a more accelerated green energy transition [5]. Within this context, GTL has emerged as a key organizational factor in promoting environmental initiatives and enhancing sustainability performance, particularly in emerging economies like Vietnam [6]. Leaders who communicate a strong environmental vision and model green values can significantly influence employees’ attitudes and behaviors toward the environment.

## 3 Data Description

The data for this study were collected on-site between October 2024 and February 2025, using a combination of convenience and snowball sampling methods. Initially, employees from conveniently accessible petroleum retail outlets in Northern Vietnam were invited to participate. After completing the questionnaire, these participants were encouraged to refer colleagues from the same or nearby outlets who met the eligibility criteria. This combined on-site approach enabled access to a geographically dispersed workforce while ensuring data authenticity and contextual accuracy. The data were collected and organized into two primary documents detailing the dataset’s structure and purpose. The first file, “Dataset.csv,” contains quantitative responses from employees in Vietnam’s petroleum retail sector, formatted for statistical analysis and theoretical modeling. This dataset includes 347 valid responses covering key variables: Green Transformational Leadership, Green Work Engagement, Meaningful Work, and Employees’ Green Behavior. The second file, “Questionnaire.docx,” comprises the complete set of survey items used for data collection. A total of 500 employees who met the study’s screening criteria were invited to participate in the survey. Ultimately, 410 responses were received, resulting in an 82% response rate. After data cleaning procedures to ensure completeness and accuracy, 347 valid responses were retained for final analysis. Each component of the dataset serves a distinct analytical purpose. The questionnaire items on Green Transformational Leadership, Green Work Engagement, Meaningful Work, and Employees’ Green Behavior provide the core measures used to examine the relationships. Demographic variables such as age, tenure, and job position are included to support subgroup or control analyses. Validity checks, covering internal consistency, convergent, and discriminant validity, ensure that the measurement model meets reliability standards for structural modeling. Together, these components form a coherent dataset that enables replication, secondary analyses, and cross-sectoral comparison, directly supporting the study’s aim of understanding how leadership practices influence employees’ pro-environmental behaviors in Vietnam’s petroleum retail sector. The descriptive demographic of respondents is presented in Table 1.

**Table 1:** Demographics of participants.

Characteristics	Code	Categories	N	(%)
Age	Q1	18–25	58	16.71
		26–36	112	32.28
		37–47	136	39.19
		> 47	41	11.82
Gender	Q2	Male	138	39.77
		Female	209	60.23
Education	Q3	High school and below	212	61.1
		University/College	96	27.67
		Master and above	39	11.24
Monthly income	Q4	Less than $300	58	16.71
		$301-$500	112	32.28
		$501-$700	136	39.19
		> $700	41	11.82
Marital status	Q5	Single	113	32.56
		Married	234	67.44

***Note:*** During the survey period, 1 USD was approximately equivalent to 26,000 VND.

Each construct in the study was carefully adapted from established literature to suit the specific context of this research. Green Transformational Leadership was measured using a six-item scale adapted from Chen and Chang [7]. Green Work Engagement was assessed using a six-item instrument derived from Aboramadan [8]. Meaningful Work was evaluated using a six-item scale originally developed by May, Gilson and Harter [9]. Employees’ Green Behavior was measured using a six-item scale based on the work of De Roeck and Farooq [10]. All constructs were assessed using a five-point Likert scale ranging from 1 (“strongly disagree”) to 5 (“strongly agree”), allowing for a nuanced analysis of the interrelationships among the variables influencing Employees’ Green Behavior. The mean scores for the scale items ranged from 2.752 to 3.605. Detailed questionnaire items are provided in Table 2. Comprehensive statistical analyses were conducted to assess the quality and distribution of the responses.

**Table 2:** Measurement scale.

Constructs	Items	Description
Green Transformational Leadership (GTL)	GTL1	The leader of the green product development project inspires the project members with the environmental plans
	GTL2	The leader of the green product development project provides a clear environmental vision for the project members to follow
	GTL3	The leader of the green product development project gets the project members to work together for the same environmental goals
	GTL4	The leader of the green product development project encourages the project members to achieve the environmental goals
	GTL5	The leader of the green product development project acts with considering environmental beliefs of the project members
	GTL6	The leader of the green product development project stimulates the project members to think about green ideas.
Employees’ Green Behavior (EGB)	EGB1	I adequately complete assigned duties in environmentally friendly ways
	EGB2	I fulfill responsibilities specified in my job description in environmentally friendly ways
	EGB3	I perform job tasks that are expected from me in environmentally friendly ways
	EGB4	I take a chance to get actively involved in environmental protection at work
	EGB5	I take initiatives to act in environmentally friendly ways at work
	EGB6	I adequately complete assigned duties in environmentally friendly ways
Green Work Engagement (GWE)	GWE1	My environmental-related tasks inspire me
	GWE2	I am proud of the environmental work that I do
	GWE3	I am immersed in my environmental work
	GWE4	I am enthusiastic about my environmental tasks at my job
	GWE5	I feel happy when I am working intensely on environmental tasks
	GWE6	With environmental tasks at my job, I feel bursting with energy
Meaningful Work (MW)	MW1	The work I do on this job is very important to me.
	MW2	My job activities are personally meaningful to me.
	MW3	The work I do on this job is worthwhile.
	MW4	My job activities are significant to me.
	MW5	The work I do on this job is meaningful to me.
	MW6	I feel that the work I do on my job is valuable.

**Source(s):** Authors’ work.

These analyses included the computation of mean, standard deviation, and variance to assess response variability. Additionally, skewness and kurtosis were calculated to evaluate the shape of the data distribution. These statistical analyses are essential for validating the measurement reliability and ensuring the robustness of the dataset. Summary statistics for each item, including central tendency and distribution measures, are presented in Table 3.

**Table 3:** Descriptive statistics of construct items.

	Mean	Median	Min	Max	Standard Deviation	Excess Kurtosis	Skewness
GTL1	3.559	4	1	5	0.758	0.205	−0.42
GTL2	3.542	4	1	5	0.807	0.068	−0.268
GTL3	3.605	4	1	5	0.757	0.169	−0.20
GTL4	3.533	4	1	5	0.721	0.318	−0.325
GTL5	3.573	4	1	5	0.746	0.036	−0.337
GTL6	3.582	4	1	5	0.790	0.178	−0.202
GWE1	3.084	3	1	5	0.721	−0.403	−0.173
GWE2	3.179	3	1	5	0.702	−0.102	−0.114
GWE3	3.098	3	1	5	0.733	−0.452	−0.067
GWE4	3.104	3	1	5	0.740	−0.327	0.003
GWE5	3.11	3	1	5	0.732	0.007	−0.218
GWE6	3.086	3	1	4	0.690	−0.358	−0.274
EGB1	2.804	3	1	4	0.753	−0.244	−0.229
EGB2	2.793	3	1	4	0.722	−0.135	−0.217
EGB3	2.752	3	1	4	0.768	−0.312	−0.195
EGB4	2.764	3	1	4	0.786	−0.482	−0.127
EGB5	2.752	3	1	4	0.783	−0.286	−0.257
EGB6	2.787	3	1	4	0.786	−0.278	−0.284
MW1	3.406	3	1	5	0.720	0.102	−0.275
MW2	3.386	3	1	5	0.725	−0.077	−0.054
MW3	3.401	3	2	5	0.758	−0.422	−0.099
MW4	3.415	3	2	5	0.760	−0.378	−0.027
MW5	3.444	3	2	5	0.724	−0.267	0.014
MW6	3.412	3	2	5	0.720	−0.283	−0.015

**Source(s):** Authors’ work.

Table 4 presents the statistics used to evaluate the constructs: Green Transformational Leadership, Green Work Engagement, Meaningful Work, and Employees’ Green Behavior. Outer loadings ranged from 0.714 to 0.773, indicating strong correlations between the observed items and their respective constructs. All outer loading values exceeded the recommended threshold of 0.70, supporting indicator reliability [11]. This validation emphasizes the accuracy of the items used to measure the construct. According to [12] the Variance Inflation Factor (VIF) values ​​in the external model range from 1.511 to 1.769, indicating small correlations between predictors in the construct; these do not adversely affect the model. Cronbach’s alpha (CA), a measure of a questionnaire's internal consistency or reliability, displays values ​​above 0.80 [11], confirming high reliability. All constructs' composite reliability (CR) values exceeded 0.80, indicating strong internal consistency. In addition, the Average Variance Extracted (AVE) values for each construct were above the recommended threshold of 0.50, confirming adequate convergent validity [11].

**Table 4:** Result of reliability and convergent validity.

	Outer loading	VIF	CA	CR	AVE
EGB1	0.727	1.535	0.843	0.884	0.561
EGB2	0.746	1.639			
EGB3	0.721	1.511			
EGB4	0.768	1.766			
EGB5	0.773	1.769			
EGB6	0.756	1.697			
GTL1	0.765	1.729	0.844	0.885	0.562
GTL2	0.768	1.679			
GTL3	0.739	1.609			
GTL4	0.742	1.666			
GTL5	0.740	1.575			
GTL6	0.741	1.628			
GWE1	0.751	1.655	0.839	0.882	0.554
GWE2	0.727	1.532			
GWE3	0.728	1.566			
GWE4	0.756	1.673			
GWE5	0.770	1.738			
GWE6	0.734	1.551			
MW1	0.756	1.643	0.830	0.876	0.541
MW2	0.734	1.585			
MW3	0.765	1.704			
MW4	0.722	1.538			
MW5	0.721	1.525			
MW6	0.714	1.524			

**Source(s):** Authors’ work.

**Fig. 1:**
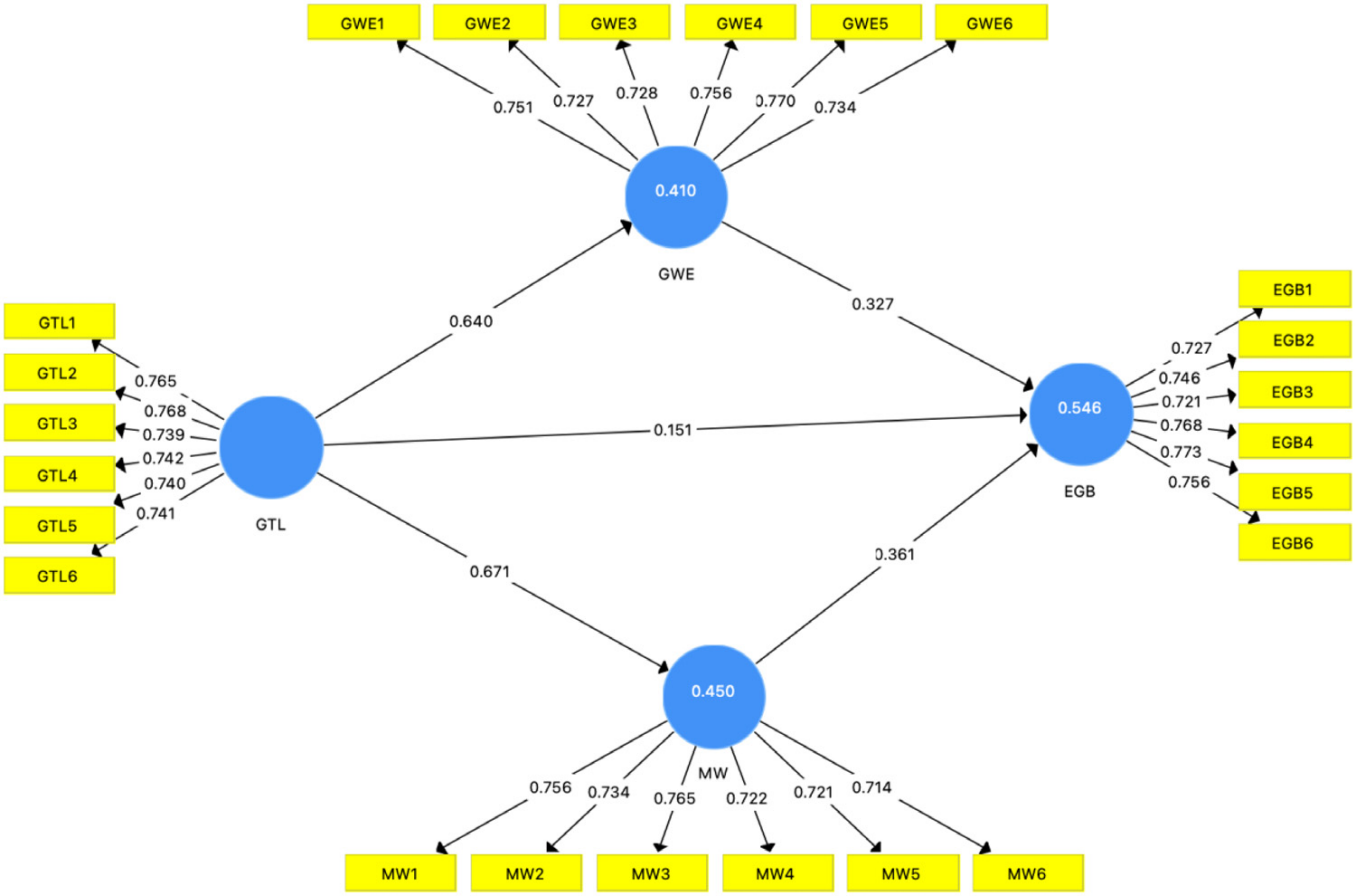
The PLS-SEM result.

Table 5 presents the values of the Fornell-Larcker criterion [13] and the Heterotrait-Monotrait ratio (HTMT). The result confirmed the discriminant validity of all constructs with HTMT values below 0.9 [14], and the square root of AVE for each construct is higher than its highest correlation with other constructs [11]. These analyses collectively substantiate the reliability and validity of our study. According to PLS-SEM algorithms, VIF values with inner model below 2.109 are considered acceptable for unbiased research tools [12]. Table 6 confirms that common method bias is not a significant issue. The Q² values were greater than zero (Employees’ Green Behavior = 0.301, Green Work Engagement = 0.223, and Meaningful Work = 0.239), indicating that the dataset demonstrates acceptable predictive relevance. Additionally, Fig. 1 illustrates the result of PLS-SEM.

**Table 5:** Discriminant validity.

Fornell-Larcker criterion				
	EGB	GTL	GWE	MW
EGB	0.749			
GTL	0.602	0.749		
GWE	0.654	0.640	0.744	
MW	0.671	0.671	0.639	0.736
Heterotrait-Monotrait Ratio (HTMT)				
	EGB	GTL	GWE	MW
EGB				
GTL	0.708			
GWE	0.775	0.758		
MW	0.801	0.799	0.765	

**Source(s):** Authors’ work.

**Table 6:** Collinearity statistic – inner model.

	EGB	GTL	GWE	MW
EGB				
GTL	2.109		1.000	1.000
GWE	1.960			
MW	2.102			

Source(s): Authors’ work.

## 4 Experimental Design, Materials and Methods

This research adopted a quantitative research design, grounded in established literature. Data were collected through direct responses to a structured survey questionnaire. To ensure the clarity and validity of the instrument, a pilot test was conducted with 50 participants. Feedback from this preliminary phase was used to refine the questionnaire's wording, structure, and layout, enhancing its clarity and overall effectiveness for the main data collection. Pilot respondents confirmed a clear understanding of the survey items. The finalized questionnaire consisted of two main sections: The first section gathered demographic information from respondents. The second section focused on the core variables of the study, which were measured using established scales adopted from prior literature. Green transformational leadership is “the behaviors of leaders who motivate followers to achieve environmental goals and inspire them to perform beyond expected levels of environmental performance” [7]. Green work engagement is conceptualized as a positive, fulfilling, and work-related psychological state characterized by vigor, dedication, and absorption in tasks associated with green tasks [8,15]. Meaningful work is defined as ‘the degree to which the employee experiences the job as one which is generally meaningful, valuable, and worthwhile [16], and Employees’ green behavior can be defined as the behaviors demonstrated by employees that have a beneficial effect on the environment [17]. All items in this section were rated using a five-point Likert scale, ranging from 1 (“strongly disagree”) to 5 (“strongly agree”), allowing for a nuanced assessment of participants’ perceptions and attitudes toward the measured constructs. To ensure the quality and reliability of the collected data, several screening procedures were conducted prior to the main analysis. First, the dataset was checked for missing values, outliers, and response consistency. As recommended by Hair et al [11], responses with >15% missing items (i.e., four or more unanswered questions out of 24 measurement items) were excluded, while cases with <5% missing values were treated using mean substitution. Second, all questionnaires were reviewed for straight-lining and inconsistent patterns to ensure response quality. To ensure reproducibility and facilitate data reuse, all analytical materials have been deposited in the Mendeley Data repository. The repository includes the raw dataset (“Dataset.csv”), the full survey instrument (“Questionnaire.docx”), the code book, the Algorithm result, the bootstrapping result, the blindfolding result, and the export project file used to perform the statistical analyses. A comprehensive README file accompanies these materials, providing definitions for all variables. These materials ensure full transparency, enabling other researchers to reproduce the analytical procedures and reuse the dataset for comparative, longitudinal, or model extension studies.

The study surveyed a total of 347 participants. In terms of age distribution, the largest group of respondents were between 37 and 47 years old (39.19%), followed by those aged 26 to 36 (32.28%), 18 to 25 (16.71%), and over 47 years (11.82%). Regarding gender, a majority of participants were female (60.23%), while males accounted for 39.77%. In terms of education level, 61.1% of respondents had a high school education or below, 27.67% held a university or college degree, and 11.24% had completed a master’s degree or higher. For monthly income, 39.19% reported earning between $501 and $700, 32.28% earned between $301 and $500, 16.71% earned less than $300, and 11.82% earned more than $700. As for marital status, 67.44% of participants were married, while 32.56% were single. A meticulously conducted in-person survey was administered to employees in the petroleum retail sector across Northern Vietnam, covering major cities and provinces such as Hanoi, Hai Phong, Quang Ninh…

After data collection, a thorough review was conducted to ensure accuracy and completeness. The dataset was then analyzed using SmartPLS 3, focusing on evaluating construct validity and reliability. Key metrics assessed included AVE, CR, and CA, all of which met the recommended thresholds, confirming acceptable levels of internal consistency and convergent validity. The AVE results indicated that a substantial portion of the variance in each construct was explained by its respective indicators, rather than by measurement error. VIF values were examined and found to be within acceptable limits to assess multicollinearity, ensuring the constructs were sufficiently distinct. Discriminant validity was further evaluated using both the Fornell-Larcker criterion and the Heterotrait-Monotrait ratio, both of which supported the distinctiveness of the constructs.

## Limitations

This study, which investigates the relationship between Green Transformational Leadership and Employees’ Green Behavior within Vietnam’s petroleum retail sector, is subject to certain limitations. This study employed a combination of convenience and snowball sampling to collect 347 responses, a practical and widely accepted approach in social science research, but this non-probability method may limit external validity. The generalizability of the findings is restricted due to the specific cultural and industry context in which the research was conducted. As a result, caution should be exercised when attempting to apply these results to other sectors or geographic regions with differing organizational norms, leadership practices, or environmental policies. Future research could adopt probability-based or stratified sampling methods to enhance representativeness and validate these findings across broader contexts.

## Ethics Statement

Participants were fully informed of the study's objectives and scope prior to participation, and informed consent was obtained accordingly. Participation was voluntary, and respondents were assured that their anonymity and confidentiality would be strictly maintained. The survey included a cover letter outlining these ethical assurances, noting that completing the questionnaire implied consent. No personally identifiable information was collected, and all responses were anonymized using a unique coding system to protect participant identity. As the study did not involve vulnerable populations and presented minimal risk to participants, approval from an Institutional Review Board (IRB) was not required. The research adhered to the ethical principles of the Declaration of Helsinki, and all participants were aged 18 years or older.

## CRediT Author Statement

**Thuy Phuong Thi Do**: Conceptualization, Supervision, Methodology, Software, Data curation, Investigation, review & editing; **Ha Thu Nguyen**: Conceptualization, Methodology, Software, Validation, Formal analysis; **Tuan Dinh Tran**: Conceptualization, Methodology, Software, Data curation, Investigation, Original draft preparation, Writing.

## Data Availability

Mendeley DataData_GTL_EGB (Original data)

## References

[1] Farrukh M., Ansari N., Raza A., Wu Y., Wang H. (2022). Fostering employee's pro-environmental behavior through green transformational leadership, green human resource management and environmental knowledge. Technol. Forecast. Soc. Chang..

[2] Lathabhavan R., Kaur S. (2023). Promoting green employee behaviour from the lens of green transformational leadership. Leadersh. Organ. Dev. J..

[3] Mukhtar A., Mahmood S., Naeem M., Khan K.I. (2025). I feel green with my leader: when and how green transformational leadership influences employees’ green behavior. Int. J. Ethics Syst..

[4] Giang T. (2024). For the petroleum market to develop stably, transparently and effectively. https://nhandan.vn/de-thi-truong-xang-dau-phat-trien-on-dinh-minh-bach-va-hieu-qua-post821766.html.

[5] (2024). EOR-NZ, Vietnam Energy outlook report (2024) - pathways to net zero. https://www.vietdata.vn/post/vietnam-energy-outlook-report-2024-pathways-to-net-zero#:~:text=The%20Vietnam%20Energy%20Outlook%20Report,a%20faster%20pace%20than%20previously.

[6] Van H.V., Hoai T.T., Minh N.N., Nguyen N.P. (2023). Green transformational leadership and green mindfulness as contributors to green innovation and environmental performance: evidence from manufacturing firms in Vietnam. Sage Open..

[7] Chen Y.S., Chang C.H. (2013). The determinants of green product development performance: green dynamic capabilities, green transformational leadership, and green creativity. J. Bus. Ethics.

[8] Aboramadan M. (2022). The effect of green HRM on employee green behaviors in higher education: the mediating mechanism of green work engagement. Int. J. Organ. Anal..

[9] May D.R., Gilson R.L., Harter L.M. (2004). The psychological conditions of meaningfulness, safety and availability and the engagement of the human spirit at work. J. Occup. Organ. Psychol..

[10] De Roeck K., Farooq O. (2018). Corporate Social responsibility and ethical leadership: investigating their interactive effect on employees’ Socially responsible behaviors. J. Bus. Ethics.

[11] Hair J.F., Hult G.T.M., Ringle C.M., Sarstedt M., Danks N.P., Ray S. (2021).

[12] Kock N. (2015). Common method bias in PLS-SEM: a full collinearity assessment approach. Int. J. e-Collab. (ijec).

[13] Fornell C., Larcker D.F. (1981). Structural equation models with unobservable variables and measurement error: algebra and statistics. J. Mark. Res..

[14] Henseler J., Ringle C.M., Sarstedt M. (2015). A new criterion for assessing discriminant validity in variance-based structural equation modeling. J. Acad. Mark. Sci..

[15] Schaufeli W.B., Salanova M., González-romá V., Bakker A.B. (2002). The measurement of engagement and burnout: a two sample confirmatory factor analytic approach. J. Happiness. Stud..

[16] Hackman J.R., Oldham G.R. (1975). Development of the job diagnostic survey. J. Appl. Psychol..

[17] Norton T.A., Parker S.L., Zacher H., Ashkanasy N.M. (2015). Employee green behavior: a theoretical framework, multilevel review, and future research agenda. Organ Env..

